# Glycemic Variability Percentage: A Novel Method for Assessing Glycemic Variability from Continuous Glucose Monitor Data

**DOI:** 10.1089/dia.2017.0187

**Published:** 2018-01-01

**Authors:** Thomas A. Peyser, Andrew K. Balo, Bruce A. Buckingham, Irl B. Hirsch, Arturo Garcia

**Affiliations:** ^1^ Consultant, Menlo Park, California.; ^2^ Dexcom, Inc., San Diego, California.; ^3^ Department of Pediatric Endocrinology, Stanford University, Stanford, California.; ^4^ Department of Medicine, Division of Metabolism, Endocrinology and Nutrition, University of Washington, Seattle, Washington.

**Keywords:** Glycemic variability, Continuous glucose monitoring, Artificial pancreas

## Abstract

***Background:*** High levels of glycemic variability are still observed in most patients with diabetes with severe insulin deficiency. Glycemic variability may be an important risk factor for acute and chronic complications. Despite its clinical importance, there is no consensus on the optimum method for characterizing glycemic variability.

***Method:*** We developed a simple new metric, the glycemic variability percentage (GVP), to assess glycemic variability by analyzing the length of the continuous glucose monitoring (CGM) temporal trace normalized to the duration under evaluation. The GVP is similar to other recently proposed glycemic variability metrics, the distance traveled, and the mean absolute glucose (MAG) change. We compared results from distance traveled, MAG, GVP, standard deviation (SD), and coefficient of variation (CV) applied to simulated CGM traces accentuating the difference between amplitude and frequency of oscillations. The GVP metric was also applied to data from clinical studies for the Dexcom G4 Platinum CGM in subjects without diabetes, with type 2 diabetes, and with type 1 diabetes (adults, adolescents, and children).

***Results:*** In contrast to other metrics, such as CV and SD, the distance traveled, MAG, and GVP all captured both the amplitude and frequency of glucose oscillations. The GVP metric was also able to differentiate between diabetic and nondiabetic subjects and between subjects with diabetes with low, moderate, and high glycemic variability based on interquartile analysis.

***Conclusion:*** A new metric for the assessment of glycemic variability has been shown to capture glycemic variability due to fluctuations in both the amplitude and frequency of glucose given by CGM data.

## Introduction

Glycemic variability is a well-recognized problem in the day-to-day management of both type 1 and type 2 diabetes. Patients with diabetes who achieve mean glucose values in the euglycemic range may still be at risk for short- and long-term complications from hypoglycemia and hyperglycemia if they have high levels of glycemic variability. There is an extensive literature on glycemic variability, but numerous authors have noted the lack of consensus about the appropriate metrics for characterizing it clinically.^1,2^

In 1970, Service et al. proposed a metric for assessing glycemic variability induced by meal-time glucose excursions, the mean amplitude of glycemic excursions (MAGE), using glycemic excursions in excess of one standard deviation (SD) above the mean.^3^ Kovatchev et al. identified high glycemic variability in an article published in 2003 as a risk factor for severe hypoglycemia.^4^ In 2005, Hirsch and Brownlee hypothesized that higher levels of glycemic variability may have been responsible for the higher risk for microvascular complications among patients in the Diabetes Control and Complications Trial (DCCT) control group with reduced glycosylated hemoglobin (A1C).^5^ Rizzo in 2010 found that high glycemic variability was associated with cognitive impairment in elderly patients with type 2 diabetes.^6^ Penckofer et al. in 2012 reported that high glycemic variability was associated with physical and emotional distress and noted that “glycemic variability may be associated with lower quality of life and negative moods”.^7^ Soupal et al. in 2014 found that high glucose variability in subjects with type 1 diabetes was associated with greater incidence of microvascular complications regardless of glycemic control.^8^ Smith-Palmer and coworkers published the results of a systematic literature review in 2014 in which they found strong evidence for an association between high glycemic variability and microvascular complications in patients with type 2 diabetes.^8,9^

The prevalence of high levels of glycemic variability has been an important incidental finding in virtually all clinical studies with continuous glucose monitoring (CGM) systems. Garg and coworkers found high levels of glycemic variability in an early study with a first-generation CGM system, the Dexcom STS.^10^ Interestingly, they observed a decrease in glycemic variability over a short period of time in a subset of patients using the system for less than 10 days. Indeed, it is reasonable to expect that the successful use of a CGM system should result in reduced glycemic variability. Patients who experience reduced glycemic variability as a result of using a CGM device may be more likely to continue to use the technology, whereas patients who still struggle with high levels of glycemic variability may be at risk for discontinuing use of the technology.

New pharmacologic treatments and medical devices may also hold promise for reducing glycemic variability in patients with diabetes. Wang et al. in 2010 suggested that leptin could be used as an adjunct to insulin to reduce glycemic variability in subjects with type 1 diabetes.^11^ More recently, Sands et al. reported that the use of sotagliflozin, a dual SGLT1 and SGLT2 inhibitor, resulted in improved glycemic control in subjects with type 1 diabetes, as measured by mean glucose, incidence of hypoglycemia, insulin utilization, and several common metrics of glycemic variability.^12^ The recent approval by the U.S. Food and Drug Administration of the first artificial pancreas device system (the Medtronic 670G) has raised hope that new devices may improve glycemic control and reduce glycemic variability.^13^ New metrics of glycemic variability may be needed to assess improvements in glycemic control and reductions in glycemic variability from evolving pharmacological treatment and medical devices in diabetes.

Despite the importance of glycemic variability in both type 1 and type 2 diabetes, there is no universally accepted current metric for the assessment of glycemic variability. A recent review article on this subject noted “(t)he definition of glucose variability, however, remains a challenge due primarily to the difficulty of measuring it and lack of consensus on the best approach to be taken”.^1^ Several groups have developed computerized schemes for calculating the mean amplitude of glycemic excursions (MAGE).^14,15^ Sechterberger et al. noted, however, that computerized protocols for calculating MAGE often did not produce the same results.^16^ Rodbard has written extensively about the different methods in current use for calculating glycemic variability.^17–20^ In a commentary published in 2012 on the challenges of measuring glycemic variability, Rodbard noted nine separate issues, including the uncertainty and ambiguity regarding the choice of a single metric for glycemic variability.^18^

Cameron et al. raised similar issues in their review of extant methods for calculating glycemic variability writing “(t)he matter remains unresolved due in no small part to the number of methods used to measure glycemic variability and a lack of agreement as to what metric constitutes the ‘gold standard’ for glycemic variability”.^21^ Saisho and coworkers conducted a detailed analysis comparing different metrics for glycemic variability based on CGM data and suggested that there was no single best metric, but rather that physicians and researchers needed to understand the strengths and weaknesses of the different metrics relative to their clinical objectives.^22^ LeFloch and Kessler have defined a glucose fluctuation index based on root-mean square calculation of successive CGM data points that they contrast with other metrics, such as SD, which measure the dispersion of glucose values, but ignore the temporal ordering of points.^23^

We have proposed a simple metric that can be used by clinicians to rapidly assess the glycemic variability status of patients and thereby identify those patients with continued high levels of glycemic variability, who might benefit from additional interaction with healthcare staff for further education on the optimum use of CGM devices. The new metric, the glycemic variability percentage (GVP), can provide a quantitative measurement of glycemic variability over a given interval of time by analyzing the length of the CGM temporal trace normalized to the duration under evaluation.

The GVP is an intuitive topological measure similar to the distance traveled metric proposed by Marling et al.^24^ and the mean absolute glucose (MAG) change proposed by Hermanides et al.^25^ The distance traveled method is the sum of the absolute difference in glucose levels from successive CGM measurements over a given interval of time. MAG is a sum of all the absolute changes in glucose normalized by the time over which the measurements were made.^26^ The distance traveled, MAG change, and GVP all capture both the amplitude and frequency of glucose oscillations and may give different results compared with other accepted metrics for glycemic variability such as coefficient of variation (CV) and SD.

## Methods

Although the GVP metric is similar to MAG and the distance traveled, GVP may be easier to understand conceptually and easier to use clinically because the results are expressed as a percentage compared with the minimum line length for a given duration. The GVP method calculates the length of line from CGM data by using a trigonometric analysis of the data. In addition, the concept of a GVP relative to a norm allows for easier comparison of glycemic variability between different data sets.

The GVP is based on the fact that the length of the continuous glucose monitor temporal trace over a given interval of time depends on the degree of glycemic variability: temporal traces with high glycemic variability have greater lengths than traces with low glycemic variability. Mathematically, this is similar to the problem posed by Mandelbrot regarding the proper measurement of the coastline of Great Britain or other coastal areas with a high degree of tortuosity.^27^

**FIG. 1.**
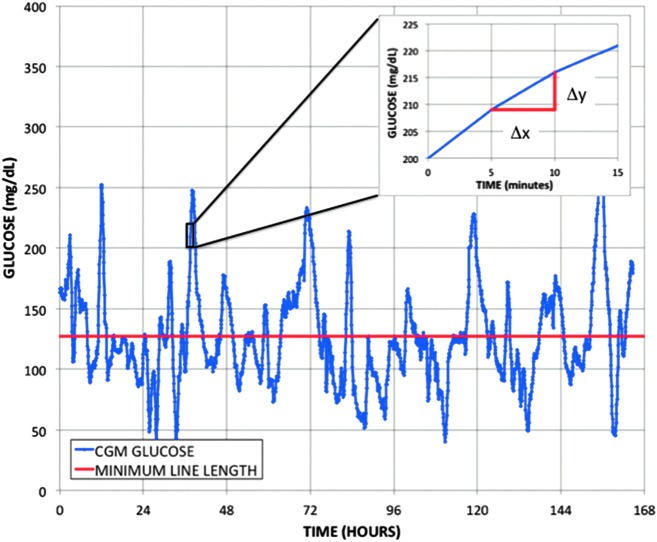
Graphical representation of the GVP defined as the length of the CGM temporal trace L (*blue*) normalized to the duration of time under study L_o_ (*red*); *Inset*: an example of the line segment decomposition required to calculate the length of each 5-min CGM segment beginning at t = 37 h and using the Pythagorean theorem to determine one line segment length. CGM, continuous glucose monitoring; GVP, glycemic variability percentage.

The GVP is normalized by the duration of time under evaluation and, hence, represents a time-invariant metric that can be applied with equal fidelity to different intervals, for example, one day, one week, one month, or three months, of CGM data. The top graph in Figure 1 shows the temporal trace from a Dexcom continuous glucose monitor (L) along with a line representing the duration of the temporal trace (L_o_)—a straight line with zero variability. The value of the GVP in this example is 30.5%.

The GVP can be calculated from the equation for the length of a line L given by the summation over all n line elements ΔL based on decomposition into horizontal (Δx) and vertical (Δy) components and application of the Pythagorean theorem.

\begin{align*}
L = \mathop \sum \limits_{i = 1}^n \sqrt { \Delta x_i^2 + \Delta y_i^2}
\end{align*}

In this equation, the differential element Δx is defined as the time between two successive CGM system measurements and the differential element Δy is determined as the change in glucose over that time interval. This is illustrated graphically in the inset in Figure 1. GVP is the ratio of the length of the line with glycemic variability compared with the length of the line without glycemic variability. The line length defined in this study is a combination of orthogonal elements, glucose and time, and includes mixed dimensions of both glucose and time. Since the dimensions of the line length calculated from the Pythagorean theorem contains units in both glucose and time, the GVP value does contain a dependency on the unit of measure of glucose (mg/dL or mmol/L). While it is possible to scale the temporal domain to compensate for changing the units of measure from mg/dL to mmol/L, to make sure that the minimal variability line used for normalization has the correct units, we recommend performing the calculation in glucose units of mg/dL.

Each line element as defined above is approximation of the instantaneous rate of change of glucose over the measurement interval between two successive CGM values. This method is therefore related mathematically to the proposals by Kovatchev et al. and Whitelaw et al. to use the rate of change of glucose, to evaluate glycemic variability.^28,29^ The GVP, is based on a simple geometric measure, namely the length of the line L represented by the CGM trace normalized to the ideal line length L_0_ for a given temporal duration or simply

\begin{align*}
{ \rm{GVP}} \; = \; \left( {{ \rm{L}} / {{ \rm{L}}_0} - 1} \right)  \times 100
\end{align*}

where L_0_ is given by

\begin{align*}
{L_0} = \mathop \sum \limits_{i = 1}^n \Delta {x_i}
\end{align*}

The duration over which glycemic variability can be calculated with this method would be calculated the same for different intervals such as days, weeks, or months. We have chosen to express the new metric as the percentage of glycemic variability above a deviation from a straight line for the duration of time under consideration. We believe that the new metric is easily understood, easily visualized, and easily calculated.

The calculations of GVP from the CGM data were performed in Excel using a simple macro or script to automate the data analysis procedure. A sample script is available upon request from the corresponding author. The script was applied to data obtained from Dexcom CGM devices with five-minute data records. Kohnert et al. evaluated the MAG change metric in subjects with type 1 and type 2 diabetes and found good results when used with CGM at high sampling rates (once every 5 min), but expressed concern that it could be misleading with low sampling rates (once every 60 min).^30^ As expected, the sampling frequency does play a role in any estimation of glycemic variability, including the GVP metric proposed in this study. Increased sampling time will have the same effect on the underlying data as the use of a low pass filter and will reduce the apparent extent of glycemic variability. Qualitative characterization of glycemic variability using the GVP metric remains valid for slightly increased sampling intervals such as once every 10 or 15 min, but the quantitative categorization (minimal, low, moderate, and high) may need to be adjusted for the longer sampling intervals. Greater sampling intervals such as 30 or 60 min are not suitable for use with the GVP metric or for other metrics that include the temporal component of glucose oscillations.

In addition, it should be noted that this method is best suited for CGM traces with high data recording rate and a low number of data omissions such as have been reported recently with new CGM systems.^31^ The algorithm is written, however, to compensate for gaps in the CGM trace by shortening the ideal line length L_0_ by the amount of missing CGM data.

We applied the new GVP metric to data obtained from five late stage or pivotal studies for the G4 Platinum in adult subjects with diabetes (*n* = 186) and three late stage or pivotal studies for the G4 Platinum in pediatric patients (*n* = 204). The example given in Figure 1 was taken from the pivotal study for the G4 Platinum (software version 505). The length of the line given by the CGM trace L was 13,349, the normalized line length L_o_ for the duration of the data was 9670, and the GVP was 38% indicative, as discussed below, of a moderate level of glycemic variability. There were 141 subjects with type 1 diabetes and 21 subjects with type 2 diabetes in the four G4 Platinum pivotal studies. In addition, we also applied the new GVP metric to 43 volunteer subjects without diabetes in two late stage feasibility studies. The duration of each of these studies was ∼7 days; hence, the total number of data points was over 450,000 or ∼288 data points per day per subject. Data from the subjects with diabetes and the volunteer subjects without diabetes were used to characterize the variation in the GVP metric observed in actual clinical data.

We compared the new glycemic variability index proposed in this study, the GVP, with two widely used metrics for glycemic variability, the SD and CV, as well as two additional metrics that have been discussed in more specialized literature on the field (MAG and CONGA1). We performed an interquartile analysis of the data in order to characterize the distribution of glycemic variability in the data obtained from the different studies. This provided numerical criteria for associating a given value of GVP with low, moderate, or high levels of glycemic variability.

## Results

**FIG. 2.**
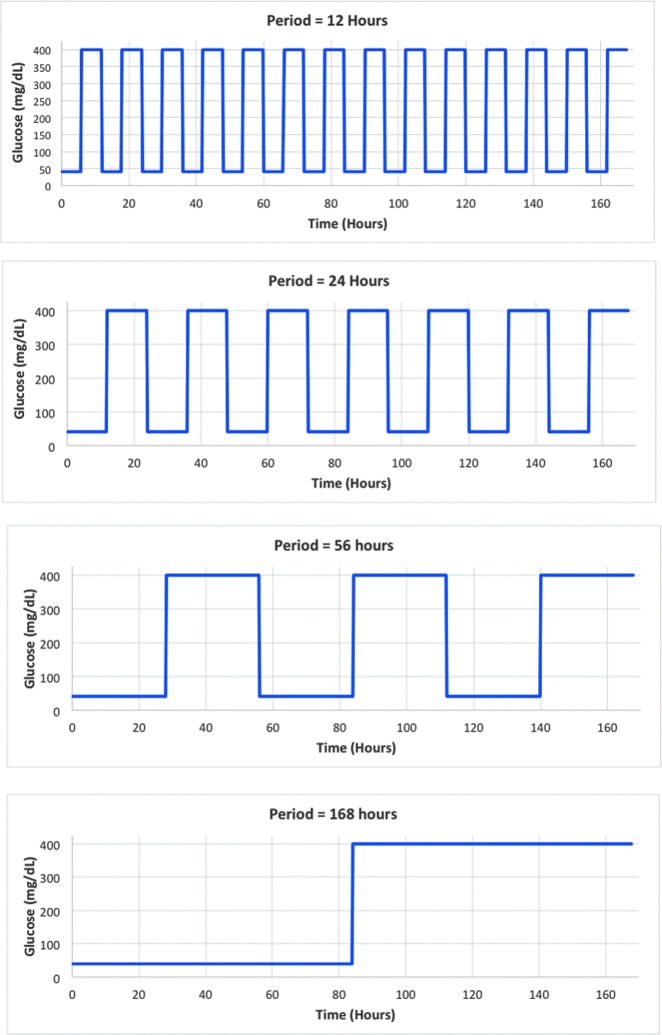
Four illustrative *square waves* with the same amplitude of oscillations, the same mean glucose, but four different periods of glycemic fluctuations (12, 24, 56, and 168 h) ranging from low glycemic variability to high glycemic variability.

Figure 2 shows four simulated CGM traces over 7 days (168 h) using square waves with the same amplitude (180 mg/dL), but different periods (12, 24, 56, and 168 h). A square wave pattern was chosen for illustrative purposes only. The minimum was 40 mg/dL, the maximum was 400 mg/dL, and the mean glucose for all four traces was 220 mg/dL. Visually, the examples with the same amplitude, but different periodicity, clearly represent different levels of variability. Four existing measures of glycemic variability were applied to this data: MAG, CONGA1, CV, and SD. The GVP metric was also applied to the data. The GVP metric was 4% for the square wave profile with a period of 84 h and 18% for the example with a period of 28 h, both indicative of a low level of glycemic variability. The GVP metric was 46% for the square wave profile with a period of 12 h and 95% for the square wave profile with a period of 6 h, both indicative of a high level of glycemic variability.

Table 1 below gives a summary of results for five metrics for glycemic variability: GVP, MAG, CONGA1, CV, and SD applied to the illustrative square wave examples in Figure 2. Four of the five metrics, excluding GVP, were calculated with the latest version of EasyGV (v9.0 R2)—an online glycemic variability resource.^32^ Results of these calculations were corroborated using a separate open source program for calculating glycemic variability metrics.^33^ In the examples shown, the mean glucose is 220 mg/dL and the SD from a continuous distribution over the same range of values is 115 mg/dL for all cases, indicating that the MAGE metric would also be unchanged for these examples.

**Table 1.** Five Glycemic Variability Metrics Applied to Simulated CGM Profiles Using Square Waves with the Same Amplitudes but Different Periods

*Period of oscillation (hours)*	*GVP (%)*	*MAG*	*CONGA1*	*CV*	*SD*
6	95	57.9	242.81	0.8184	180.04
12	46	27.9	264.01	0.8184	180.04
28	18	10.7	276.88	0.8184	180.04
84	4	2.1	283.49	0.8184	180.04

The results show that three metrics capture the difference in glycemic variability (GVP, MAG, and (CONGA), whereas two others (CV and SD) do not.

CV, coefficient of variation; GVP, glycemic variability percentage; MAG, mean absolute glucose; SD, standard deviation.

The first two metrics (GVP and MAG) are able to clearly differentiate between these four examples of glycemic variability. The numerical values of both the GVP metric and the MAG metric show that the variability is increased 4–5-fold from the 84-h period to the 28-h period. Both metrics show an ∼2.5 factor increase in glycemic variability from the 28-h period to the 12-h period and another twofold factor from the 12-h period to the 6-h period. The third metric (CONGA1) does show a difference in glycemic variability among the four examples, but it is difficult to assess the magnitude of the difference in glycemic variability from the numerical values for this metric. The additional two metrics (CV and SD), which are the most widely used tools for assessing glycemic variability, give the *same* results for all four cases.

The GVP and MAG metrics capture the visual impression from the graphs that if one could take the curves in the four examples and stretch them to their full length, the curve with the shortest periods (highest frequency) of oscillations would be longer than the curve with the longest periods (lowest frequency) of oscillations. While the simulated square wave temporal CGM traces are for mathematical demonstration purposes only, they show clearly that the different glycemic variability metrics measure different components of glycemic variability. The two glycemic variability metrics that gave the same results for all four square wave examples (CV and SD) measure the amplitude of glycemic excursions, but not the frequency, and hence may be incomplete as metrics of glycemic variability.

GVP, MAG, and CONGA1 measure both the amplitude and frequency of oscillations and hence may represent more complete measures of glycemic variability than the other metrics. Figure 2 is similar to a figure in deVries, which compared a simulated step function and a simulated sawtooth function between 5 and 10 mmol/L over a 20-h period, and found a similar result to that reported in this study, namely that the SD was unchanged between the two simulated profiles, whereas the value of the MAG metric was different by a factor of 19.^26^ In our simulations, the GVP metric is unique among the three that do capture the difference in glycemic variability in its ability to provide a physically intuitive value based on the percentage increase in glycemic variability compared with a trace with zero variability (i.e., a straight line).

**FIG. 3.**
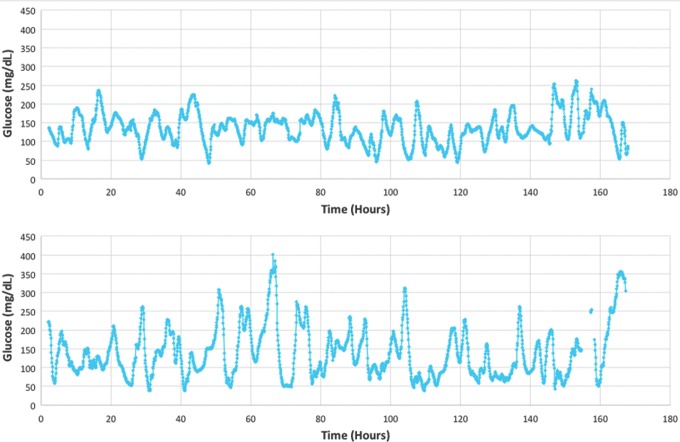
Two examples of CGM traces from a seven-day clinical study (168 h) showing low-to-moderate glycemic variability GVP = 29% (*top*) and high glycemic variability GVP = 52% (*bottom*), but approximately the same mean glucose (135 and 138 mg/dL, respectively).

In Figure 3 below, two traces are shown from research subjects in clinical studies of the Dexcom G4 Platinum CGM. Both traces have the same approximate mean glucose, but there are visually discernible differences in glycemic variability. In the first of the two traces, the mean glucose was 135 mg/dL and the GVP metric was 29%, indicating low glycemic variability as determined by interquartile analysis (see Table 3). In the second of the traces, the mean glucose was 138 mg/dL and the GVP metric was 52%, indicating high glycemic variability also as determined by interquartile analysis.

Following the methodology of Rodbard, we have performed an interquartile analysis of the glycemic variability associated with each separate study subject cohort.^19^ Analysis of clinical study data from the late stage feasibility and pivotal regulatory studies for the original G4 Platinum CGM system and the updated version (software 505) found a spectrum of glycemic variability according to the GVP metric, depending on the study subject cohort. Pediatric subjects with type 1 diabetes showed higher levels of glycemic variability than adults with type 1 diabetes. Table 2 shows the GVP values associated with study subjects without diabetes, with type 1 diabetes, with type 2 diabetes, as well as children (2–12 years old) and adolescents (13–18 years old) with type 1 diabetes.

**Table 2.** Upper Bounds of the Glycemic Variability Percentage (GVP) for Each Quartile from Interquartile Analysis of Glycemic Variability Data in Adults (>18 y/o) Without Diabetes, with Type 2 Diabetes and with Type 1 Diabetes

*GVP metric for glycemic variability*	*Adults without diabetes (%)*	*Adults with type 2 diabetes (%)*	*Adults with type 1 diabetes (%)*	*Children with type 1 diabetes (%)*	*Adolescents with type 1 diabetes (%)*
0th Pct. (min)	8.4	13.2	18.5	26.8	27.1
2.5th Pct.	8.8	14.5	22.8	39.2	30.4
25th Pct.	14.8	20.6	33.8	52.9	42.3
50th Pct.	18.2	26.0	42.3	58.1	51.9
75th Pct.	19.6	30.6	48.9	66.1	63.3
97.5th Pct.	25.3	42.6	64.0	95.0	79.3
100th Pct. (max)	28.4	44.1	69.7	95.0	112.1

Also shown is the interquartile analysis for children (<13 y/o) and adolescents (13–18 y/o) with type 1 diabetes.

Pct., percentile.

The table gives the upper bounds of the GVP for each quartile from interquartile analysis of the CGM data. In addition to the 100th percentile (maximum), the table also contains the 0th percentile (minimum), as well as the 2.5th and 97.5th percentiles. In subjects without diabetes, the upper bound of the first quartile was GVP equal to 14.8%. The upper bound of the second quartile, or the median GVP, was 18.2%. The upper bound of the GVP in the third quartile was 19.6% and the highest value of GVP in the fourth quartile was 28.4%. These results are consistent with the findings by Hill et al. calculating glycemic variability using other previously published metrics in nondiabetic subjects of various ethnicity.^34^

The lowest levels of glycemic variability were found, not surprisingly, in study subjects without diabetes. As expected, subjects with type 2 diabetes were found to have a lower level of glycemic variability than subjects with type 1 diabetes.

Adolescents with type 1 diabetes had the highest mean glycemic variability of 58.0% compared with 51.9% for children with type 1 diabetes. The median (50th percentile) glycemic variability according to the GVP metric was 42.3% for adults with type 1 diabetes, 26.1% for adults with type 2 diabetes, and 18.2% for adults without diabetes. The highest levels of glycemic variability were seen in the upper quartile of adolescents with type 1 diabetes that reached a GVP value of 112% compared with 95.0% in children with diabetes and 69.7% in adults with type 1 diabetes.

Analysis of the CGM data from nondiabetic subjects found small amounts of glycemic variability consistent with the observation by Service that, “a modest degree of variation of glycemia is characteristic of normal glucose homeostasis”.^35^ In the nondiabetic subjects, 79% of all GVP values were ≤20% and 100% of all GVP values were ≤30%. The significance of the overlap in GVP between some subjects without diabetes and some with diabetes, but minimal glycemic variability, will be discussed below. In subjects without diabetes, 95% were found to have glycemic variability as measured by GVP less than or equal to 24% compared to 38.1% of subjects with type 2 diabetes and only 3.6% of subjects with type 1 diabetes.

An additional analysis was performed in which glycemic variability according to the GVP metric was separated into four separate quartiles in subjects, for all subjects combined with diabetes (type 1 and type 2). Table 3 gives the classification of glycemic variability into four categories (minimal, low, moderate, and high) from interquartile analysis of the CGM data cohorts from subjects without diabetes, subjects with type 1 diabetes, and subjects with type 2 diabetes. The lowest quartile was associated with minimal glycemic variability for which there was significant overlap between subjects with diabetes and subjects without diabetes. The second and third quartiles were associated with low and moderate levels of glycemic variability. The fourth quartile was associated with high levels of glycemic variability. There were 19 subjects with type 1 diabetes (11.5%) in the lowest quartile of GVP (≤30%). In subjects with type 2 diabetes, there were 15 subjects in the lowest quartile (71.4%) indicative of minimal variability and 6 subjects in the second quartile (28.6%) indicative of low variability. In data from subjects with type 2 diabetes, there were no subjects in the third and fourth quartiles, indicating no case of moderate or high variability.

**Table 3.** Classification of Glycemic Variability into Four Categories from Interquartile Analysis of Multiple CGM Data Cohorts: Minimal (Non-Diabetic), Low, Moderate, and High Based on the Glycemic Variability Percentage (GVP)

*Glycemic variability*	*GVP*
Minimal (nondiabetic)	≤20
Low (first quartile diabetic)	≤30
Moderate (second and third quartile diabetic)	30–50
High (fourth quartile diabetic)	>50

**FIG. 4.**
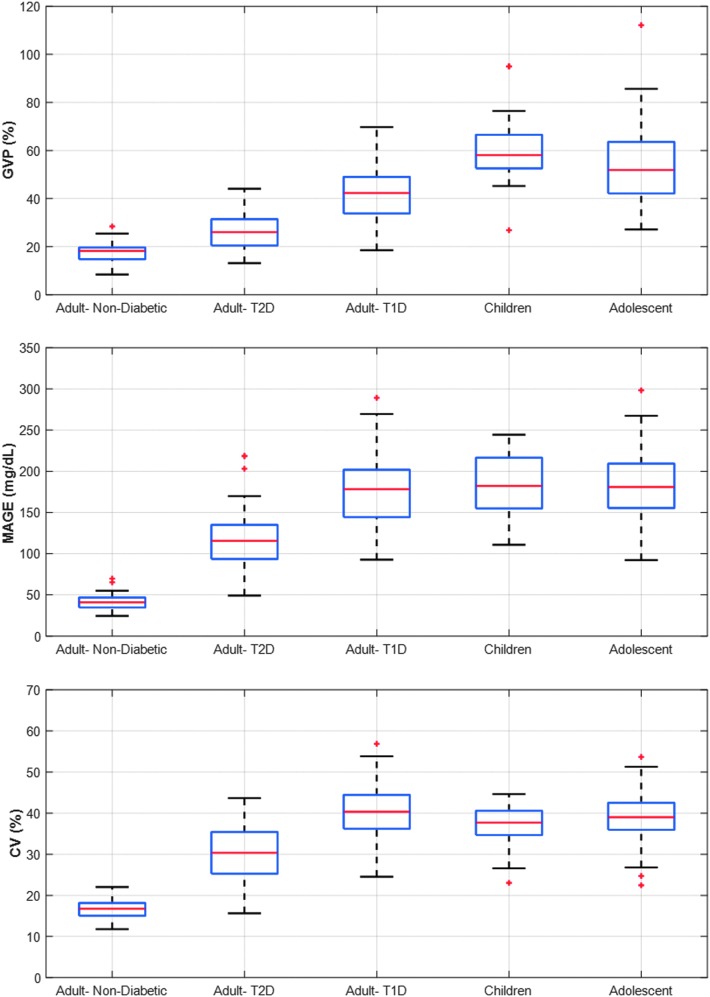
Comparison of interquartile analysis of the glycemic variability metric (GVP) and three extant metrics for glycemic variability (MAGE and CV) for five subject cohorts: adolescents (13–18 years old) and children (2–12 years old) with T1D, adults with T1D, adults with T2D, and adults without diabetes. (+) are considered outliers since they are 1.5*IQ below the 25^th^ Percentile or 1.5*IQ above 75^th^ Percentile where IQ is the interquartile range.” CV, coefficient of variation; MAGE, mean amplitude of glycemic excursions; T1D, type 1 diabetes; T2D, type 2 diabetes.

The results from Table 2 above are shown graphically below in Figure 4 for each of the five subject cohorts. The top figure shows the GVP for adolescents (13–18 years old) with type 1 diabetes, children with type 1 diabetes (6–12 years old), adults with type 1 diabetes, adults with type 2 diabetes, and adults without diabetes. The additional figures show the glycemic variability in the same subject cohorts using three established metrics of glycemic variability: MAGE, CV, and SD. All four graphs in Figure 4 clearly show the difference in glycemic variability between subjects without diabetes, adults with type 2 diabetes, adults with type 1 diabetes, adolescents with type 1 diabetes, and children with type 1 diabetes. The three established metrics for glycemic variability capture the range of variability in the five different subject cohorts, but the quantitative ordering of the measured variability is different for GVP compared with MAGE, CV, and SD. Interquartile analysis for the MAGE and SD metrics applied to all five data sets is given in Supplementary Tables S1 and S2 (see Supplementary Data at http://online.liebertpub.com/doi/suppl/10.1089/dia.2017.0187).

## Discussion

The earliest metric proposed for characterizing glycemic variability, MAGE, was designed for assessment of postprandial glucose excursions above the mean glucose. The MAGE metric was originally used with a laboratory continuous blood glucose analyzer with measurements made once every 5 min, but the calculation of glycemic variability was made from the maximum values of glucose excursions associated with meals and taken approximately once every four to six hours.^3^ The MAGE metric also been used extensively with temporally discrete blood glucose measurements.^36^ Two of the more widely used mathematical metrics, the SD and the CV, are suitable for both temporally discrete and continuous data, but may be misleading when applied to certain types of data. In Figure 2 above, we showed four simulated glucose profiles exhibiting different levels of glycemic variability, for which two metrics (CV and SD) give the same numerical results in contrast to three other metrics (GVP, MAG, and CONGA1), which were able to differentiate between the four temporal profiles.

Comparison of the proposed new GVP metric with two widely accepted metrics of glycemic variability, MAGE and CV, found significant differences in the relative ordering of variability between the five subject cohorts. The highest variability measured by the GVP metric was found in the adolescent cohort with type 1 diabetes. Children with type 1 diabetes were also found to have higher variability than adults with type 1 or type 2 diabetes. By contrast, results from the MAGE and CV metrics suggest that glycemic variability is comparable in adolescents and adults with type 1 diabetes and higher in adults than in children. In addition, according to the GVP metric, adults with type 2 diabetes have markedly lower variability than children with type 1 diabetes, whereas both the MAGE and CV metrics suggest that glycemic variability is comparable between these groups. As we have shown with the interpretation of the square wave example, the GVP metric (along with MAG) measures both the amplitude and frequency of glycemic fluctuations. Since the amplitude of glycemic fluctuations may be comparable in all age cohorts with type 1 diabetes, whereas the frequency of fluctuations may differ, it is not surprising that GVP would indicate differences in glycemic variability compared with MAGE, CV, and other metrics that depend exclusively on or are heavily weighted to the amplitude of oscillations only.

There were some subjects without diabetes whose GVP values overlapped significantly with diabetic subjects classified as having minimal glycemic variability. Review of the data from the subjects without diabetes found GVP less than 20% in 79% of subjects and less than 24% in 95% of all subjects. In the upper quartile of GVP for subjects without diabetes, 33% (3/9) had impaired fasting glucose values in excess of 100 mg/dL meeting clinical criteria for prediabetes. Although speculative, in other cases of GVP greater than 20%, the elevated level of glycemic variability may indicate normal physiologic fluctuations in glucose in a subset of individuals or it may be suggestive of possible future development of prediabetes or type 2 diabetes. Additional research is needed to determine whether glycemic variability, as characterized by the GVP metric or other metrics for glycemic variability, can be used as an early indicator of impending prediabetes or type 2 diabetes before abnormal values are detected in the fasting glucose or A1C.^37^

In subjects with diabetes, glycemic variability is an important component of overall assessment of glycemic control, but it needs to be augmented by assessment of mean glucose and the incidence of hypoglycemia. In a previous article, we have proposed a composite index for overall glycemic control combining the GVP, the mean glucose, time in range, and the frequency and severity of hypoglycemic events.^38^

## Conclusions

The potential role of glycemic variability in short- and long-term diabetic complications has been widely postulated and studied by numerous authors. However, the effect of glycemic variability on quality of life and patient self-perception of personal efficacy in managing diabetes has received only limited attention to date. We believe that successful sustained use of modern CGM systems should be accompanied by measurable decreases in glycemic variability. The metric proposed in this study, the GVP, can be easily understood and readily calculated from CGM data. Although CGM utilization rates are still relatively low compared with the total population of patients who could benefit from the technology, improvements in CGM accuracy and reliability coupled with the recent approval by the FDA for the use of CGM to guide insulin dosing may dramatically increase adoption of CGM in the years ahead.

Already CGM data are being posted to cloud-based servers making it possible to run analytics automatically, which assess quantities such as glycemic variability in patients using the technology. While some patients understand intuitively how to use CGM devices to improve their glycemic control, there are others who require more extensive training and support. Clinical studies are needed to explore whether automated detection of persistent glycemic variability in new users of CGM devices may help healthcare providers better identify patients who would benefit from additional training and education on the optimum use of the technology. In a companion article, we apply the proposed GVP metric to two sets of data: an early published study on the effect of CGM use on glycemic variability and a more recently published article on a bihormonal artificial pancreas clinical study.^39^

The new glycemic variability metric proposed in this study, the GVP, may be helpful in outcome studies that include CGM as a means of correlating clinical endpoints such as frequency of severe hypoglycemia or DKA with glycemic variability. Rodbard found a strong correlation between glycemic variability as measured by CV and the incidence of hypoglycemia.^19^ Jin et al. also found a strong association between relative glycemic variability as measured by CV and the incidence of hypoglycemia, but not between absolute glycemic variability as measured by SD and hypoglycemia.^40^ Similarly, Qu and coworkers found that the rate of hypoglycemia in patients with type 2 diabetes on insulin was strongly correlated with relative glycemic variability as measured by intraday CV, but not with absolute glycemic variability as measured with MAGE.^41^ Monnier et al. have suggested that there may be identifiable thresholds separating clinically acceptable and unacceptable levels of glycemic variability such as values of CV greater than 36%.^42^ Additional research is needed to determine if there are similar identifiable thresholds using the GVP metric proposed in this study.

In a recent review article, Kovatchev and Cobelli noted that many extant metrics of glycemic variability are skewed to the amplitude of the oscillations and give little weight or neglect altogether the temporal component of the oscillations.^43^ This is a controversial issue, as noted in a comment by Service who argued against the inclusion of the temporal component in the assessment of glycemic variability.^44^ The new metric proposed in this study, the GVP, gives weight to both the amplitude and frequency of glucose oscillations (the temporal component) and hence, may be more useful than other available measures in correlating acute and chronic complications with glycemic variability as well as mean glucose. Application of this metric and comparison with other amplitude-based glycemic variability metrics to clinical outcome studies are needed to better understand these issues.

Finally, the new glycemic variability metric proposed in this study may also be useful as part of a composite metric to evaluate multiple dimensions of glycemic control based on CGM data alone. Composite metrics for glycemic control have been proposed previously by several researchers.^45,46^ Hirsch et al. have described a composite metric, the personal glycemic state, combining the GVP described in this study with the mean glucose, percent time in range (70–180 mg/dL), and the incidence and severity of hypoglycemia.^38^

## Supplementary Material

Supplemental data

Supplemental data
