# Supplementary material for: Glycemic Variability Percentage: A Novel Method for Assessing Glycemic Variability from Continuous Glucose Monitor Data
Source: Diabetes Technol Ther. 2018 Jan 1;20(1):6–16. doi: 10.1089/dia.2017.0187 (PMC5846572; doi:10.1089/dia.2017.0187)
Supplement: Supplemental data [file Supp_Table2.pdf]

Supplementary Table S2. Upper Bounds of the Coefficient of Variation (CV) for Each Quartile from Interquartile Analysis of Glycemic Variability data in Adults (>18 y/o) Without Diabetes, with Type 2 Diabetes and with Type 1 Diabetes

CV metric for glycemic variability	*Adults without diabetes (%)*	*Adults with type 2 diabetes (%)*	*Adults with type 1 diabetes (%)*	*Children with type 1 diabetes (%)*	*Adolescents with type 1 diabetes (%)*
0th Pct. (min)	11.8	15.6	24.5	23.0	22.4
2.5th Pct.	12.3	16.5	28.1	25.4	26.4
25th Pct.	15.0	25.4	36.2	34.8	35.9
50th Pct.	16.7	30.4	40.3	37.7	39.0
75th Pct.	18.1	35.4	44.4	40.5	42.5
97.5th Pct.	20.9	42.1	52.5	44.5	50.5
100th Pct. (max)	22.0	43.7	56.9	44.6	53.7

Also shown is the interquartile analysis for children (< 13 y/o) and adolescents (13–18 y/o) with type 1 diabetes.
CV, coefficient of variation.
